# Cases of Insanity; with Medical, Moral, and Philosophical Observations and Essays upon Them

**Published:** 1832-05

**Authors:** 


### BIBLIOGRAPHICAL NOTICE.

*Cases of Insanity; with Medical, Moral, and Philosophical, Observations, and Essays upon them.* By M. Allen, M.D. &c. Part I. Vol. I. *This first Part contains an Essay on Atmospheric, the Seasons, Diurnal, Lunar, and Planetary, Influence; with which is incorporated an Inquiry into the Causes of Epidemics, and of Cholera Morbus in particular.*—8vo. pp. 212. Swire, London.

Although we are given to understand that the continuation of this work depends on the encouragement this first part of the first volume receives, and although we learn, in the 200th page, that the author has "some very interesting cures" "to state in due course," still we cannot, in conscience, say much in favor of the present publication, and must, therefore, reserve our praises until the "very interesting cures" are stated in due course, when our commendations may come in due course also.

Whether or not it be contagious elsewhere, that cholera is contagious in "the Row" no doubt can be entertained; for scarce a book can escape, whatever be its import, from the cholera contamination. We suppose the trade guess, and shrewdly too, as to what will take, and books now-a-days are rather made to sell than read. There is fashion in all things: and even authors, it would seem, must take their subjects, or at least accommodate their titles, to the prevailing taste, and the public constitution appears at present to labour under decided symptoms of the choleric diathesis: for what else can account for a writer on Insanity filling half of his scanty pages with a huge digression on Cholera; and more especially when the materials, so far from being original, are verbatim reprints from the columns of the "Times" newspaper and from KENNEDY's work on this disease, unless it be that a name may be adopted which will arrest the public eye. It does, notwithstanding all due allowance, appear to us to be somewhat out of the due course of *authorship* to give verbatim extracts, for forty pages together, in the section on Cholera Morbus, which only occupies sixty pages in the whole, and when fifteen more out of the sixty are filled with extracts from newspapers and other sources: and this when the "Cases of Insanity, with medical, moral, and philosophical observations," are all comprised within thirty pages, and the Essay on the Influence of the Atmosphere properly only extends to sixty; for the author himself confesses, "I have made this essay on Atmospheric Influence in reality conclude at page 75, and all that follows is an appendage," and, as we would hint, an incumbrance also, which had much better have been omitted; for then room would have been left for the insertion of those interesting cures that are hinted at, and also for more copious details of the cases given, which, as they now appear, are meagre in the extreme, as may be guessed from the fact of twenty-two cases, with (as we have already observed) the medical, moral, and philosophical observations," only occupying thirty pages. Indeed, we scarcely see any other use they are of than to shew that the records have been badly kept. This, the author probably will tell us, occurred before his time, and that his registers are more satisfactorily arranged: indeed, we doubt it not; and we only regret that Dr. ALLEN did not draw upon his own resources, instead of upon Kennedy's work and epistles from the newspapers.

